# Ultrasensitive Nano-rt-iPCR for Determination of Polybrominated Diphenyl Ethers in Natural Samples

**DOI:** 10.1038/s41598-017-12339-x

**Published:** 2017-09-20

**Authors:** Xiaohan Zhang, Xianyin Ping, Huisheng Zhuang

**Affiliations:** 1 School of Environmental Science and Engineering, Shanghai Jiao Tong University, Minhang District, Shanghai, 200240 China; 2 East China Sea Fisheries Research Institute, Yangpu District, Shanghai, 200082 China

## Abstract

Extensive polybrominated diphenyl ethers (PBDEs) use has resulted in its increasingly widespread presence in the environment. PBDEs release from existing products can still persist and accumulate in the environment as well as in human and wildlife magnifying through the food web. Due to its ultra-trace amount in the environment, a novel ultrasensitive nano-rt-iPCR assay has been developed to determine polybrominated diphenyl ethers in natural samples. Numerous amino-DNA and polyclonal antibody (anti-PBDE) were immobilized onto the single-walled carbon nanotubes (SWCNTs) to form antibody-SWCNTs-DNA signal amplifier used in the proposed immunoassay system. Compared with rt-iPCR, this nano-rt-iPCR assay had a higher ratio of signal DNA, which meant higher signal measured and lower detection limit. This proposed nano-rt-iPCR assay was used to determine PBDEs in water samples ranging from 0.5 pg/L to 0.5ng/L; giving the LOD 1 pg/L. To the best of our knowledge, this nano-rt-iPCR is the most sensitive method for PBDEs detection. Because of that, this method needs no pre-concentration or extractions, using sample sizes as low as 10 µL. In general, this nano-rt-iPCR method will be a useful and potential way for batch detection of ultra-trace PBDEs in the aquatic environment.

## Introduction

Polybrominated diphenyl ethers (PBDEs) are brominated flame retardants added into commercial products, which consisting of a mixture of congeners (penta-, octa- and deca- BDEs”. Extensive PBDEs use has resulted in its increasingly widespread presence in the environment^1^, especially the lower halogenated BDEs accumulate to a greater degree than the higher ones^2^. Considering their potential to accumulate in the environment, the European Union (EU) banned the use of their commercially available mixtures (penta- and octa- BDE in 2004; deca-BDE in 2008). And the China government has controlled the use of the mixtures of PBDEs in 2006 and agreed to ban the marketing and use of penta-BDEs and octa-BDEs in 2007. Despite the bans, extensive use of PBDEs will still be a serious problem because of the release from existing products into the environment for the foreseeable future^3^. As a result, PBDEs can persist and accumulate in the environment as well as in human and wildlife^3–9^, magnifying through the food web^10^ for many years.

Commonly used methods for analysis of PBDEs are gas chromatography-mass spectrometry(GC-MS)^11^, gas chromatography negative chemical ionization–mass spectrometry (GC–NCI–MS)^12^, GC/high resolution MS (GC/HRMS)^13^ or gas chromatography-electron capture detection(GC–ECD). These methods are usually not only limited to a laboratory environment but also with complicated sample extraction or pre-concentration because of the detection limit. So, to improve the limit-of-detection and simplify the sample preparation are significant challenges in the research of analysis methods, particularly for samples with ultra-trace levels. Accurate, cost-effective and rapid immunoassays, such as enzyme-linked immunosorbent assay and real-time immuno-PCR, have provided attractive ways for the analytical chemists. Compared with conventional enzyme-linked immunosorbent assay, researches have showed that the immuno-PCR may be the most appropriate one due to its ultra-high sensitivity^14,15^ and good quantification capabilities^16^. With good merits, the rt-iPCR has been used to detect cancer^17,18^, bacterial pathogen^19^ and environmental pollutants^20,21^.

**Figure 1:**
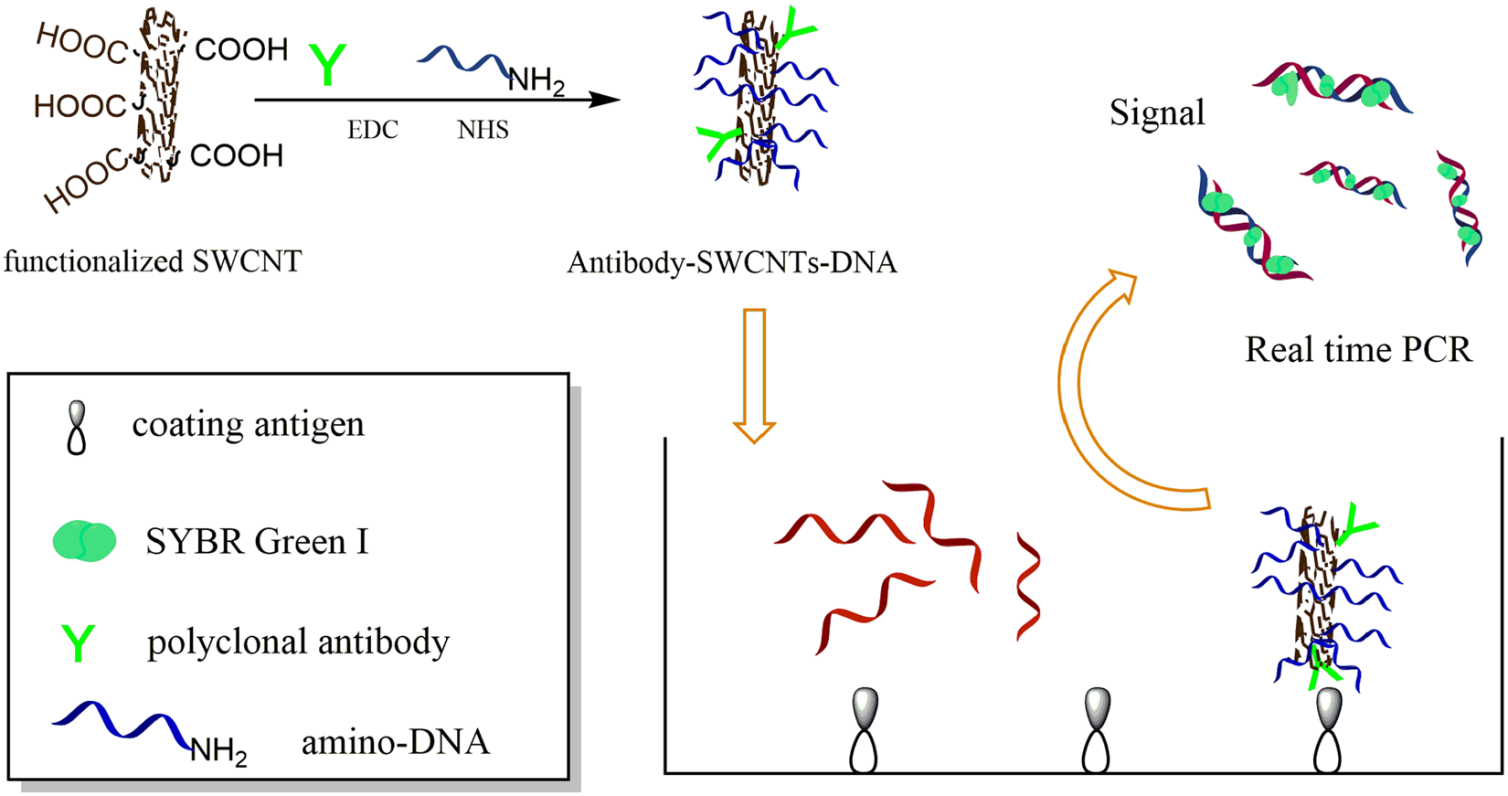
The nano-rt-iPCR principle.

In our study, a novel ultrasensitive nano-rt-iPCR assay has been developed to determine polybrominated diphenyl ethers in natural samples (The nano-rt-iPCR principles were shown in Fig. 1). Hapten, immunogen, coating antigen and polyclonal antibody were synthesized first. Numerous amino-DNA and polyclonal antibody were immobilized onto the single-walled carbon nanotubes (SWCNTs) to form SWCNTs-DNA signal amplifier used in the proposed immunoassay system. Compared with rt-iPCR, this nano-rt-iPCR assay had a higher ratio of signal DNA, which meant higher signal measured and lower detection limit. In addition, this nano-rt-iPCR assay was applied to determination of polybrominated diphenyl ethers in water without any pre-concentration or extractions using sample sizes as low as 10 µL. In general, this nano-rt-iPCR method would be a useful and potential way for batch determining the ultra-trace PBDEs in aquatic environment.

## Results and Discussion

### Preparation of hapten

**Figure 2:**
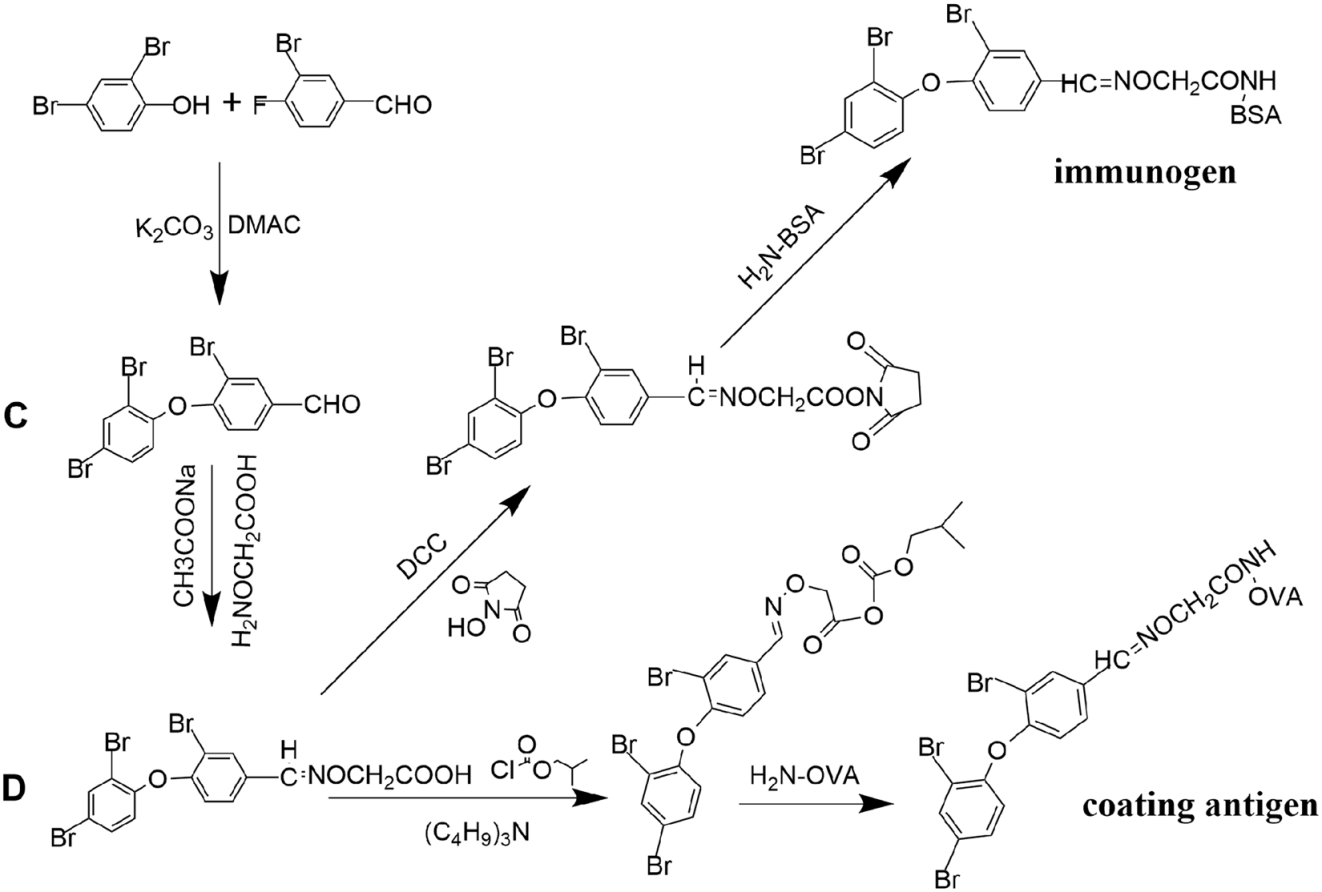
The synthetic route of antigens, immunogen and coating antigen.

Hapten was synthesized first, those procedures were illustrated in Fig. 2. The analysis of synthetic compounds were as follows: *3-Bromo-4-(2*,*4-dibromophenoxy)benzaldehyde (C):* IR(KBr, ν/cm^−1^): 3085.4(C-H, Ar-H stretching vibration),2843.3,(C-H,-CHO stretching vibration), 1691.5(C = O, stretching vibration), 1595.7,1565.5,1462.7(C = C, framework vibration), 1374.0(C-H,-CHO flexural vibration), 1243.0,1044.0(C-O-C, stretching vibration), 681.2(C-Br stretching vibration). ^1^H NMR(DMSO): 9.93(s,1H,-CHO), 8.27(s,1H,Ar-H), 8.08(s,1H, Ar-H), 7.87(d,1H,Ar-H), 7.67(d, 1H, Ar-H), 7.22(d,1H,Ar-H), 6.95(d,1H,Ar-H). *BDE-47 hapten (D):* IR(KBr, ν/cm^−1^): 3433.9(O-H, stretching vibration), 3085.2(C-H, Ar-H stretching vibration), 2975.2(C-H,-CH_2_ stretching vibration), 1691.9(C = O, stretching vibration),1595.8, 1566.7, 1463.3(C = C, framework vibration), 1637.4(C = N,stretching vibration), 1243.2, 1044.9 (C-O-C, stretching vibration), 682.2 (C–Br stretching vibration). ^1^H NMR(DMSO): 12.82(s, 1H, -COOH), 8.35(s,1H,-CH = N-), 8.03(s,1H,Ar-H), 7.98(d,1H,Ar-H),7.62(s,1H,Ar-H),7.59(s,1H,Ar-H),7.00(s,1H,Ar-H),6.96(s,1H,Ar-H),4.63(d,2H,-O-CH_2_-).

### Preparation of immunogen and coating antigen

**Figure 3:**
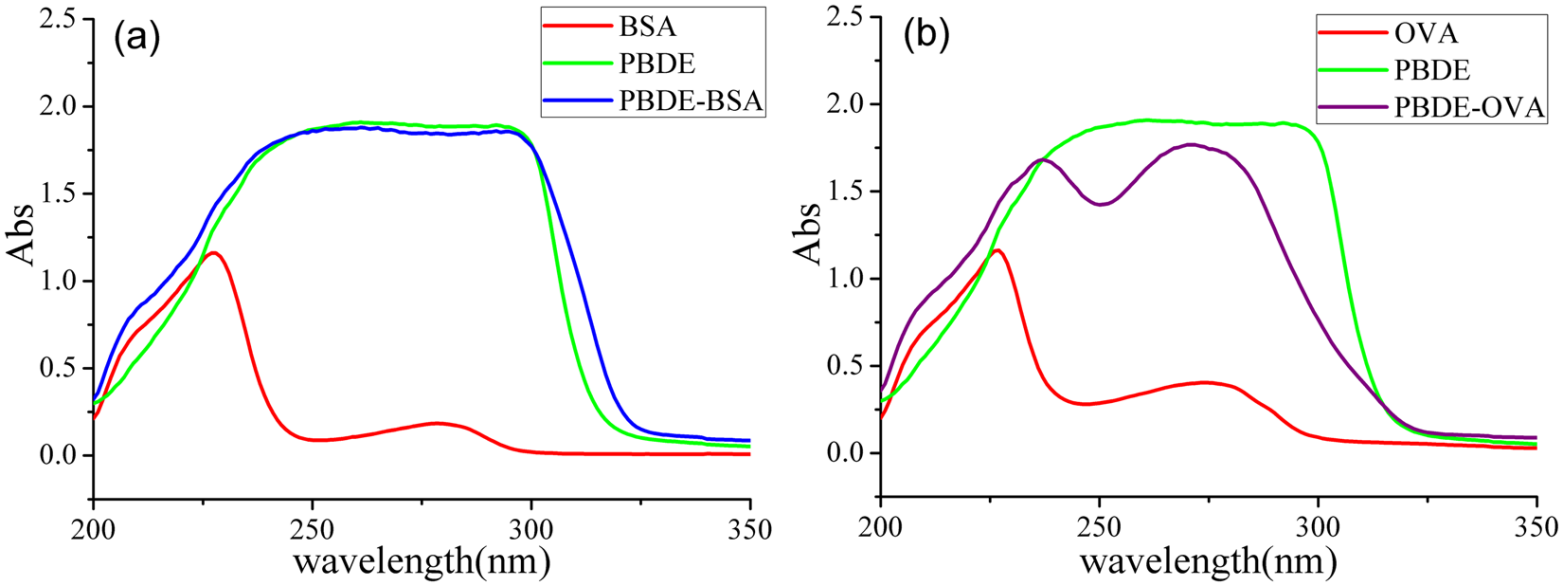
The UV spectra of PBDE hapten, protein and conjugates: (**a**) with BSA and (**b**) with OVA; absorbance value at the characteristic peak, 292 nm: OD_BSA-PBDE_ = 1.860, OD_PBDE-hapten_ = 1.893, OD_BSA_ = 0.074; 272 nm: OD_OVA-PBDE_ = 1.767, OD_PBDE_-_hapten_ = 1.893, OD_OVA_ = 0.401; C_BSA_: 0.20 g/L, C_OVA_: 0.20 g/L, and C_hapten_: 0.05 g/L; protein and conjugate were dissolved in PBS buffer; hapten was dissolved in DMSO.

For coupling BDE-47 hapten (D) with BSA or OVA, different coupling methods, were applied on BDE-47 hapten by using carboxyl group (Fig. 2). The ultraviolet-visible (UV-Vis) spectrophotometer was used for analyzing the conjugates (Fig. 3). The characteristic absorption peaks: BDE-47 hapten: 261 and 292 nm; BSA: 227 and 279 nm; OVA: 227 and 274 nm. However, for BSA-BDE-47 and OVA-BDE-47 were 292 nm and 272 nm. Successfully, the BDE-47 hapten was conjugated with BSA and OVA according to the results. Moreover, the coupling ratios of BSA-BDE-47 and OVA- BDE-47 were 31 and 15.

### Characterization of the SWCNTs-DNA amplifier

**Figure 4:**
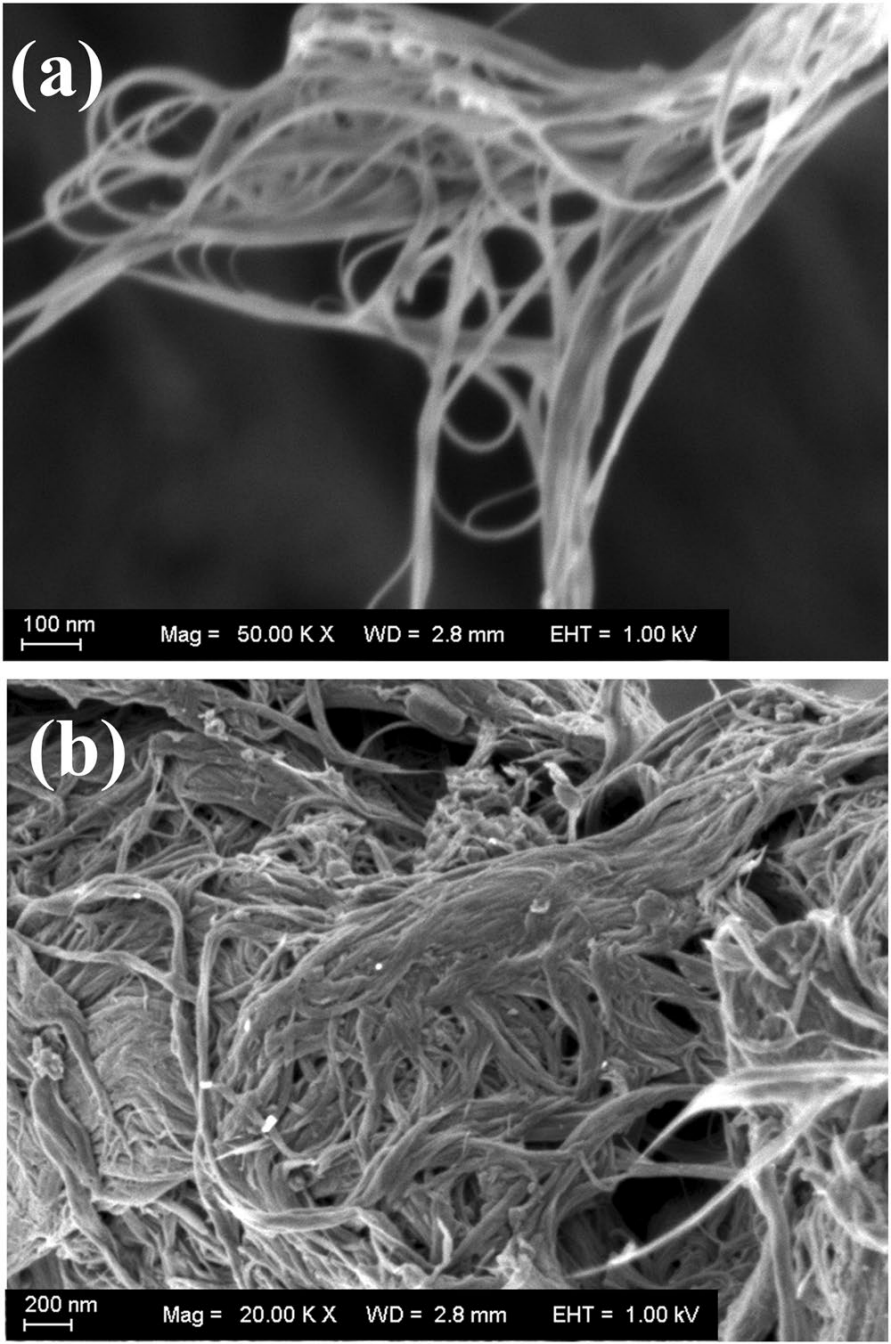
The carboxylated SWCNTs (**a**) and the SWCNTs-DNA amplifier (**b**).

Single-walled carbon nanotubes were applied to immobilize numerous amino-DNA and polyclonal antibody. In this study, scanning electron microscope (SEM) was used to image the SWCNTs before and after the conjugation. Compared with the carboxylated SWCNTs in Fig. 4(a), the thicker diameter of antibody-SWCNTs-DNA amplifier as seen in Fig. 4(b) could imply the binding of amino-DNA and polyclonal antibody to SWCNTs.

### Optimized conditions for nano-rt-iPCR

**Figure 5:**
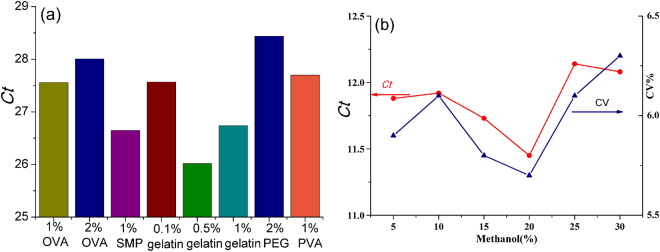
Optimized conditions of the immunoassay: (**a**) the blocking reagent, (**b**) organic solvents’ concentrations.

Several parameters were optimized to establish the nano-rt-iPCR method. Firstly, the concentrations of SWCNTs-DNA amplifier and coating antigen were optimized by using the checkerboard assay. Coomassie Blue staining method was used for determining the concentrations of BDE-OVA (1.94 mg/mL) and polyclonal antibody-SWCNTs-DNA amplifier (0.82 mg/mL). According to the checkerboard assay, the optimal concentration of BDE-OVA was 4.85 μg/mL, and the pAb-CNTs-DNA amplifier was at 1:200 dilution(10 < *Ct*_min_ < 15). The *Ct* results were shown in Table 1. Different blocking solutions, such as SMP (1%), PEG 20 000(1%), PVA (1%), gelatin (0.1%, 0.5% and 1%) and OVA (1% and 2%) in PBS, were compared. The better blocking reagent was, the larger *Ct* value should achieve. As shown in Fig. 5(a), the maximal *Ct* (2% PEG, 28.44) was achieved. For other blocking solutions, the values were as follows: 0.1% gelatin (26.74), 0.5% gelatin (26.02), 1% gelatin (27.57), 1% OVA (27.56), 1% SMP (28.01), 1% PEG 20 000 (28.44), and 1% PVA (27.70). Therefore, the blocking solution (2% PEG) was selected. Considering the influence of organic solvents’ concentrations, different amounts of ethanol were added. As shown in Fig. 5(b), the results indicated that sensitivity was significantly affected by the amount of organic solvents in the buffer. The lowest *Ct* value was determined when 20% methanol (v/v) in PBS buffer, hence 20% methanol(v/v) was selected.

**Table 1:** The optimum concentrations for nano-rt-iPCR.

Dilutions of SWCNTs-DNA amplifier	BDE-OVA concentration(μg/mL)				
	9.7	4.85	2.43	1.21	0.61
25	7.15	7.36	7.66	7.92	8.13
50	8.02	7.99	8.14	8.21	8.22
100	8.61	9.38	9.62	9.74	10.45
200	10.96	10.35	11.37	11.63	12.11
400	11.98	12.89	13.48	14.87	15.02

### Cross-Reactivity

Organic compounds, such as 2, 4, 6-Tribromophenol, TBBPA, BDE-28, BDE-100, PCB-8, PCB-28, PCB-47 were selected as analogues to BDE-47. Cross-reactivity values (%) were shown in Table 2. Only BDE-28 showed relatively high CR (21.2%), whereas others were low. According to the cross-reactivity, this antibody not only had high affinity to BDE-47 but also BDE-28.

**Table 2:** Cross-reactivity.

Common name	Structure	IC_50_ (ng/mL)	CR (%)
PCB-47		50.91	1.1
PCB-28		93.34	0.6
PCB-8		186.67	0.3
BDE-100		11.91	4.7
BDE-28		2.64	21.2
TBBPA		31.11	1.8
2,4,6-Tribromophenol		34.91	1.6
BDE-47		0.56	100

### Sensitivity and standard curve

**Figure 6:**
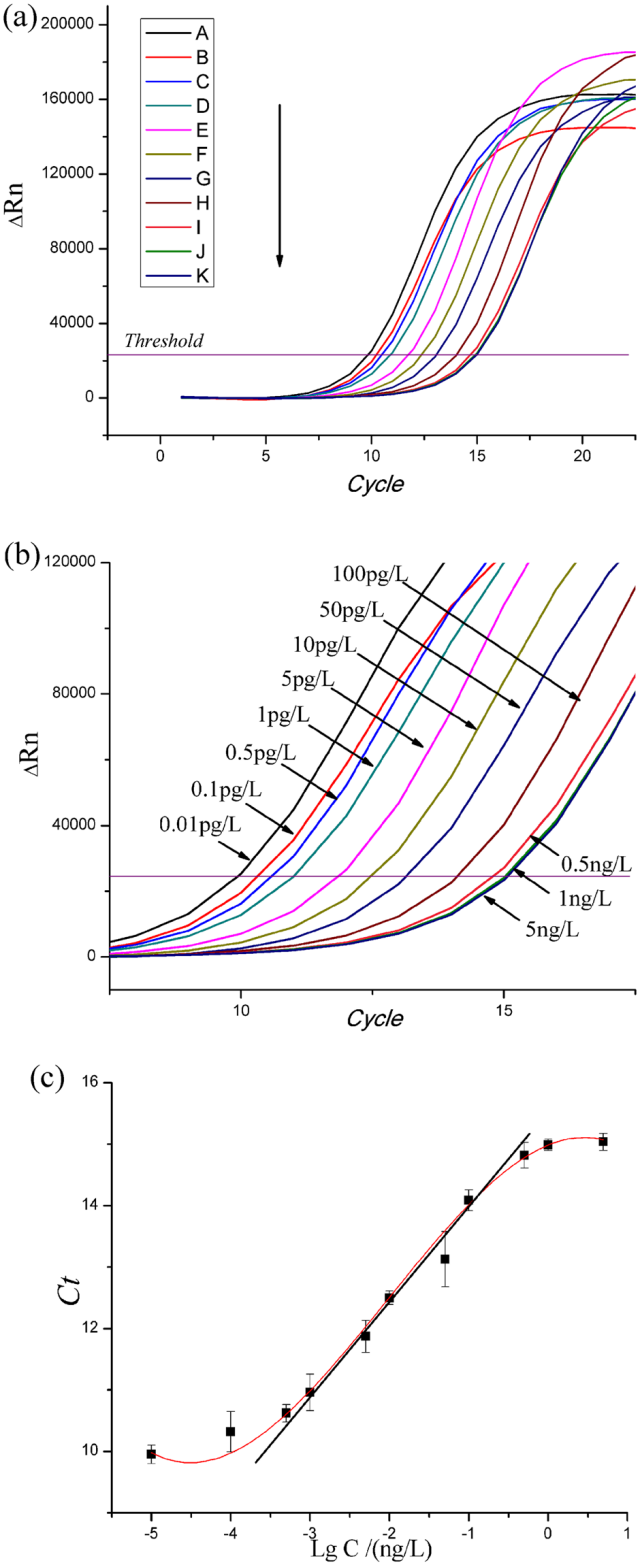
The amplification curves (**a**), (**b**) and standard curve (**c**) of nano-rt-iPCR for detecting PBDEs. The concentrations were A:0.01 pg/L, B:0.1 pg/L, C:0.5 pg/L, D:1 pg/L, E:5 pg/L, F:10 pg/L, G:50 pg/L, H:100 pg/L, I:0.5ng/L, J:1ng/L and K:5ng/L.

Under optimal conditions, the correlation coefficient of the BDE-47 standard curve was 0.986; the slope was 1.428; the intercept was 15.261; with the linear regression equation Y = 1.428 × lgC + 15.261. The linear working range was 0.5 pg/L–0.5 ng/L, and the standard curve of nano-rt-iPCR was shown in Fig. 6. Under optimal conditions, the LOD (3.3σ/S) of nano-rt-iPCR assay was 1 pg/L (the *Ct* value of blank was 10.35 ± 0.43).

### Application to real samples and recovery tests

For evaluating the precision of this nano-rt-iPCR assay, the concentrations of BDE-47 in water samples were also determined on a GC-ECD. In general, the results of nano-rt-iPCR assay were slightly higher (Table 3). This might be caused by the different samples’ preparation methods or the antibodies’ cross-reactivity for other organic compounds presented in water sample, such as BDE-28, etc. Considering that the BDE-47 was at much higher concentrations compared with other lower halogenated BDEs, so the results were acceptable. The repeatability of the immunoassay of PBDEs was assessed by recovery tests. For the recoveries, each sample was tested six times, and the results are described in Table 4. Average recovery rates of this immunoassay were 92.4% to 110.2%, and the CVs were 3.2% to 8.1%.

**Table 3:** Concentrations of PBDEs in water by the nano-rt-iPCR and GC-ECD.

Samples	Concentration(mean±SD)(μg/L) (n = 3)	
	The nano-rt-iPCR	GC-ECD
1	0.0122 ± 0.035	<LOD
2	0.0196 ± 0.083	<LOD
3	0.1160 ± 0.032	0.1086 ± 0.024
4	0.0324 ± 0.101	0.0346 ± 0.015
5	0.0258 ± 0.037	<LOD
6	0.0016 ± 0.075	<LOD

**Table 4:** Recoveries of PBDEs in water by the nano-rt-iPCR.

Samples	Sample Concentration (μg/L)	Spiked level (μg/L)	Tested concentration (μg/L)	Average recovery% ±CV%(n = 6)
1	0.0122	0.01	0.0219	97.0±3.2
		0.02	0.0313	95.5 ±4.6
		0.10	0.1165	104.3 ±8.1
3	0.1160	0.05	0.1622	92.4 ±7.5
		0.10	0.2241	110.2 ±6.3
		0.20	0.3290	106.5 ±3.4

### Comparison of correlated methods

This nano-rt-iPCR assay was developed by combining the real time immuno-PCR with single-walled CNTs immobilized numerous signal DNA. Compared with the commonly used immunoassay (the ELISA method developed in our previous work^22,23^, with the limit of detection in μg/L), the sensitivity of nano-rt-iPCR assay was significantly improved with the limit of detection in pg/L. Comparing with conventional real-time immuno-PCR^24^, this nano-rt-iPCR method was more convenient and less procedures. Moreover, compared with other nanoparticle based assay (magnetic beads used to separate particles, antibody-antigen complex, and DNA before the determination), nano-rt-iPCR made the SWCNTs-DNA amplifier immobilized on the PCR tube directly via the reaction between antibody and antigen, which simplified the assay and saved time. In addition, due to the preassembled SWCNTs-DNA amplifier, the ratio of antibody and signal DNA immobilized on the CNTs was improved, which meant higher sensitivity. Thus, no pre-concentration or extractions were needed due to its sensitivity and stability. As a result, this nano-rt-iPCR assay would be useful for ultrasensitive determination of PBDEs in environmental studies.

### Conclusion

First, this study developed a nano-rt-iPCR assay for PBDEs determination in water. Hapten, immunogen and coating antigen, were synthesized for the first time. After the immunization, the polyclonal antibody was obtained. Second, the SWCNTs were used to immobilize numerous amino-DNA and polyclonal antibody to form SWCNTs-DNA amplifier, which were used as signal-amplifier in the proposed immunoassay system. Compared with real time immuno-PCR, this nano-rt-iPCR had a higher ratio of signal DNA, which meant higher sensitivity. This proposed nano-rt-iPCR assay was used to determine PBDEs in water samples in the range from 0.5 pg/L to 0.5ng/L, giving the LOD 1 pg/L. Moreover, due to the sensitivity and specificity, this nano-rt-iPCR needed no pre-concentration or extractions, using sample sizes as low as 10 µL. In general, this nano-rt-iPCR method would be a useful and potential way for batch detection of ultratrace amounts of PBDEs in the aquatic environment.

## Materials and Methods

### Instruments

The^1^H Nuclear Magnetic Resonance (NMR) Spectrometer: Ascend^TM^ 400 MHz instrument (Bruker, Switzerland) with DMSO-D6 solution. Fourier transforms infrared spectrometry: Nicolet 6700 instrument (Thermo, USA). Scanning electron microscopy: ZEISS-Merlin compact (ZEISS, Germany). Quantitative real-time PCR: Step One Plus Real-Time PCR system (Applied Biosystems, USA) with 8 Strip Real-time PCR Tubes. The setup of the PCR instrument’s parameters were: 35 cycles of 20 s at 95 °C, 20 s at 57 °C, and 20 s at 72 °C, with the final extension of 3 min at 72 °C, after which a melting procedure is continued. The ultrapure water: Milli-Q system (Millipore, Bedford, MA, USA).

### Reagents and solutions

The standard and single-walled carbon nanotubes, carboxylic acid functionalized, 95%, diam.: <2 nm, length: 1–3 μm were purchased from J&K Chemical Technology (Beijing, China). Silica gel (100–200 mesh) was purchased from Shanghai Sanpont (China). Complete Freund’s adjuvant, ovalbumin (OVA), Bovine serum albumin (BSA), dichloromethane, n-hexane, Tween-20, N-hydroxysuccinimide (NHS), 1-(3-Dimethylaminopropyl)-3-ethyl carbodiimide hydrochloride(EDC), dimethyl sulfoxide (DMSO), O-(carboxymethyl)hydroxylamine hemihydrochloride, methanol, Coomassie Brilliant Blue G250 and anhydrous sodium acetate were purchased from Sinopharm, China. Analytical grade was required for all the reagents. SGExcel FastSYBR Mixture (SYBR Green I), amino-DNA and primers were purchased from Shanghai Sangon Biotech, China. The sequences of the amino-DNA and sscDNA were shown in Table 5; the PCR mixture system was shown in Table 6. Phosphate buffered saline (PBS buffer): Na_2_HPO_4_ 10 mmol/L; KCl 2.7 mmol/L; KH_2_PO_4_ 2 mmol/L; NaCl 137 mmol/L. PBST: 0.05% Tween-20 in PBS.

**Table 5:** The sequences of DNA and primers.

Name	Sequences
Amino-DNA	5′-AGCGAGGAAGCGGAAGAGCGCCCAATACGCAAACCGCCTC—(T)_10_-(CH_2_)_3_-NH_2_-3′
sscDNA	5′-GAGGCGGTTTGCGTATTGGGCGCTCTTCCGCTTCCTCGCT-3′
Primer1	5′-GAGGCGGTTTGCGTATTG-3′
Primer2	5′-AGCGAGGAAGCGGAAGAG-3′

**Table 6:** The PCR mixture system.

Reagents	50 μL reaction mixture system
SGExcel FastSYBR Mixture	25 μL
Forward Primer, 5 µM	5 μL
Reverse Primer, 5 µM	5 μL
RNase-Free ddH_2_O	up to 50 μL

### Preparation of hapten

The synthetic procedures of **C** were according to Marsh^25^ and Yeager^26,27^, the synthetic procedures of **D** were according to Lee^28^ and Chen^29^. *3-Bromo-4-(2,4-dibromophenoxy)benzaldehyde (C):* 2,4-Dibromophenol (2.0 g, 8.0 mmol) and 3-bromo-4-fluorobenzaldehyde(1.62 g, 8.0 mmol) in DMAC (15 m L), anhydrous potassium carbonate(1.22 g, 8.8 mmol), heated and stirring for 6 h. The mixture was cooled, and water (50 mL) and CH_2_Cl_2_ (30 mL) were added. The CH_2_Cl_2_ phase was collected and dried (MgSO_4_). After evaporation, the yellow organic residue was purified (silica column chromatography; Eluent: n-hexane/CH_2_Cl_2_, 1:1) and light yellow oil (1.84 g) was obtained with the yield of 52.9%. *BDE-47 hapten (D)*: 3-Bromo-4-(2,4-dibromophenoxy)benzaldehyde(0.1305 g,0.3 mmol) in CH_2_Cl_2_ (0.3 mL), O-(carbonxymethyl)hydroxylamine hemihydrochloride (0.039 g, 0.36 mmol), anhydrous sodium acetate(0.03 g, 0.36 mmol) were added into methanol(20 mL), stirring with a reaction time of 3–4 h. After evaporation, the residue was dissolved into water(10 mL) with NaOH aqueous adjusting pH to 14, then washed three times with CH_2_Cl_2_(30 ml). The pH of water layer was adjusted to 1 by adding HCl aqueous, then extracted four times with CH_2_Cl_2_(30 ml). The CH_2_Cl_2_ phase was collected and dried (MgSO_4_). The filtrate was crystallized by rotary evaporation. Light yellow solid BDE-47 hapten (0.104 g) was obtained with the yield of 68.3%.

### Preparation of immunogen and coating antigen

For coupling BDE-47 hapten (D) with BSA or OVA, different coupling methods, were applied on BDE-47 hapten by using carboxyl group. The details were as follows^22,30^: For the activated ester method, BDE-47 hapten, NHS and DCC were dissolved in 0.5 m L DMAC (molar ratio of BDE-47 hapten to NHS and DCC 1:1.2:1.2) and stirred for 6 h. BSA (10 mg/mL) in PBS mixture was prepared; the obtained organic supernate was added slowly and stirred at 4 °C overnight. PBS (0.01 M, pH 7.4) was used for dialysis of the suspension for 3 d. For the mixed anhydride method, 50 mg (0.1 mmol) of BDE-47 hapten was dissolved in 250 μL DMAC. Isobutylamine and n-butyl chloroformate ester were added and stirred for 3–4 h (molar ratio of BDE-47 hapten to isobutylamine and n-butyl chloroformate ester was 1:1.2:1.2). After the reaction, the suspension was added dropwise into OVA solution (10 mg/mL) and stirred at 4 ^◦^C overnight. PBS (0.01 M, pH 7.4) was used for dialysis of the suspension for 3 d.

### Preparation of polyclonal antibody

Hapten-BSA (1 mg/mL, Freund’s adjuvant emulsified; complete/incomplete; 1:1; v/v), was injected into the New Zealand white rabbits (male, 2.0–2.5 kg) at intervals of 2–3 weeks for the immunization. After the successful immunization, the antiserum was separated from the rabbit blood (anti-PBDEs antibodies inside); and the antiserum was purified. The immunization was same as in our previous work^30^.

### Preparation of SWCNTs amplifier

Signal amplifier was prepared based on literatures^31–33^ with a slight modification. The antibody and amino-DNA were simultaneous loading onto the functionalized SWCNTs via EDC coupling. Details were as follow: SWCNTs (1.0 mg) were dispersed in PBS buffer (2 mL) and stirred 20 min. After that, 1 mL pH 6.0 MES buffer of EDC (400 mM) and NHS (100 mM) mixture was added. The mixture solution was centrifuged; the pellet was then redispersed in MES buffer (1 mL). Amino-DNA and antibodies were added to the mixture solution and stirred for 4–5 h. Several washing or centrifugation steps were required to remove the excess reagents. The antibody-SWCNTs-DNA amplifier was diluted by PBST immediately before use.

### Sample preparation

**Figure 7:**
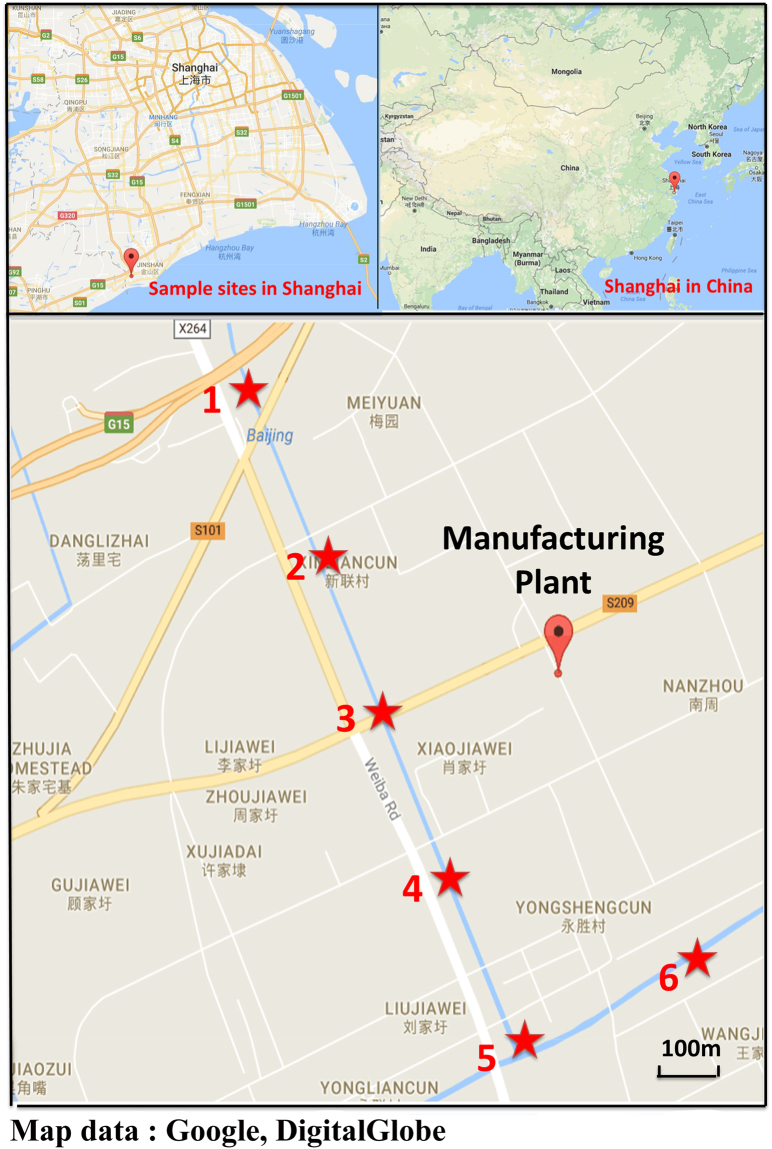
Sampling sites. Map data: google, DigitalGlobe.

The surface water samples were collected by clean glass bottles in May 2017. The sampling site and detailed information were shown in Fig. 7. All the glassware used in this study was pre-cleaned with methanol and then washed with ethanol three times. For this immunoassay, no pre-concentrations or extractions were needed; the samples were stored at 4 °C in a dark place. PBS buffer containing 40% methanol was added into samples (1:1; v: v) before analysis; the samples analyzed in triplicate. For the recovery study, PBDE was analyzed six times. For the GC-ECD, 500 mL of water sample was filtrated first (0.45μm filter) and then loaded onto the solid phase extraction (SPE) cartridge. The SPE cartridge was preconditioned and washed with 40% methanol (5 mL) first; then eluted with methanol (9 mL). Finally, the eluent was evaporated under N_2_ to 200μL.

### Ethics statements

The experiments carried out on animals were in accordance with the institutional and national guidelines. Also, all experimental protocols were approved by animal experimental center, School of Agriculture and Biology, Shanghai Jiao Tong University.
