# Author Correction: Ultrasensitive Nano-rt-iPCR for Determination of Polybrominated Diphenyl Ethers in Natural Samples

**DOI:** 10.1038/s41598-018-27431-z

**Published:** 2018-06-20

**Authors:** Xiaohan Zhang, Xianyin Ping, Huisheng Zhuang

**Affiliations:** 1 School of Environmental Science and Engineering, Shanghai Jiao Tong University, Minhang District, Shanghai 200240 China; 2 East China Sea Fisheries Research Institute, Yangpu District, Shanghai 200082 China

Correction to: *Scientific Reports* 10.1038/s41598-017-12339-x, published online 20 September 2017

In the original version of this Article, there were errors in Affiliation 1 which was incorrectly listed as ‘School of Environment Science and Technology, Shanghai Jiao Tong University, Minhang District, Shanghai, 200240, China.’ The correct affiliation is listed below:

School of Environmental Science and Engineering, Shanghai Jiao Tong University, Minhang District, Shanghai, 200240, China.

These errors have now been corrected in the PDF and HTML versions of the Article.

